# Impact of Landfill Gas Exposure on Vegetation in Engineered Landfill Biocover Systems Implemented to Minimize Fugitive Methane Emissions from Landfills

**DOI:** 10.3390/ijerph20054448

**Published:** 2023-03-02

**Authors:** Dinu S. Attalage, J. Patrick A. Hettiaratchi, Angus Chu, Dinesh Pokhrel, Poornima A. Jayasinghe

**Affiliations:** 1 Department of Geoscience, University of Calgary, 2500 University Drive, NW, Calgary, AB T2N 1N4, Canada; 2 Department of Civil Engineering, Center for Environmental Engineering Research and Education (CEERE), University of Calgary, 2500 University Drive, NW, Calgary, AB T2N 1N4, Canada

**Keywords:** methane oxidation, landfill gas, plant stress, fugitive methane emissions, landfill biocover systems

## Abstract

Engineered landfill biocovers (LBCs) minimize the escape of methane into the atmosphere through biological oxidation. Vegetation plays a crucial role in LBCs and can suffer from hypoxia caused by the displacement of root-zone oxygen due to landfill gas and competition for oxygen with methanotrophic bacteria. To investigate the impact of methane gas on vegetation growth, we conducted an outdoor experiment using eight vegetated flow-through columns filled with a 45 cm mixture of 70% topsoil and 30% compost, planted with three types of vegetation: native grass blend, Japanese millet, and alfalfa. The experiment included three control columns and five columns exposed to methane, as loading rates gradually increased from 75 to 845 gCH_4_/m^2^/d over a period of 65 days. At the highest flux, we observed a reduction of 51%, 31%, and 19% in plant height, and 35%, 25%, and 17% in root length in native grass, Japanese millet, and alfalfa, respectively. The column gas profiles indicated that oxygen concentrations were below the levels required for healthy plant growth, which explains the stunted growth observed in the plants used in this experiment. Overall, the experimental results demonstrate that methane gas has a significant impact on the growth of vegetation used in LBCs.

## 1. Introduction

Despite the practices of reducing, recycling, and reusing, considerable amounts of municipal solid waste (MSW) end up in sanitary landfills. MSW in sanitary landfills undergoes biochemical reactions and produces landfill gas (LFG), primarily comprised of the greenhouse gasses, methane (CH_4_) and carbon dioxide (CO_2_) [1,2,3]. Engineered landfill biosystems, such as landfill biocovers (LBCs), bio-windows, and methane biofilters populated with methanotrophic bacteria, or methanotrophs, with the capability of oxidizing CH_4_ can potentially be used to reduce the fugitive methane emissions to the atmosphere [4,5]. Vegetation is incorporated in LBC design for erosion control, improvement of the aesthetic value, and to encourage evapotranspiration [6,7]. Furthermore, the presence of vegetation in LBCs alters the water balance and microbial community dynamics [8,9]. The condition of the vegetation on LBCs impacts the growth and activity of methanotrophs, and the vegetation type and coverage become determining factors in biological CH_4_ oxidation [10,11]. Therefore, maintenance of a healthy growth of vegetation on LBCs is vital for efficient biological CH_4_ oxidation. However, because of the potential hostile environment presented by LBCs, maintaining healthy vegetation is a serious challenge.

Several researchers have reported that LFG can severely inhibit plant growth on landfill surfaces [12,13]. In a study involving grass, vines, and herbs, a negative correlation between the vegetation cover on landfills and LFG emissions was observed [14]. Stunted growth of corn (*Zea mays* Strurtev.) and sweet potato (*Ipomea batata* L.) was observed when the root zones of the plants were exposed to LFG [15]. Tao et al. [6] observed that species diversity of plants grown on restored landfill surfaces decreased over time, indicating the effect of LFG on vegetation growth is plant species specific. Other researchers have aimed at understanding the causes and mechanisms of plant distress encountered at landfills [6,15,16,17,18,19,20]. Toxicity of LFG, depleted oxygen (O_2_), soil CO_2_, thin layer of cover soil, nutrient deficiencies, low water holding capacity of cover material, high soil temperatures, high soil compaction, and the use of sensitive plants have been identified as key reasons for limited vegetation growth at landfill sites [21]. Early research has shown that an O_2_ level above 10% in the soil-gas phase of the root zone is needed for the growth of most plants, and plant growth can be impacted at levels below 10–15% [19,22]. The escape of significant amounts of LFG across the landfill surface has the potential to displace O_2_ in the root zone, thereby lowering the O_2_ levels below the threshold levels [13,18]. However, Gilman et al. [16] found that certain plant species were able to withstand low O_2_ tension in the soil and tolerated landfill conditions better than others. In addition to causing hypoxia that retards the growth of vegetation, the components of LFG themselves could also impact vegetation growth [15]. Past researchers have noted that although CH_4_ is not phytotoxic, elevated levels of CO_2_ can cause damage to roots even in the presence of adequate amounts of O_2_ in the root zone [15,20].

Most past studies have focused on gaining an understanding of plant growth on impacted soil surfaces, including landfill final covers, and they have identified two factors that cause the stunted growth of plants: the lack of O_2_ and the presence of high levels of CO_2_ in the root zone. A key aspect that has not been studied in detail is the contribution of soil methanotrophy to plant stress. Biological oxidation of CH_4_ in LFG by methanotrophs consumes O_2_ in the root zone and also produces CO_2_ [23], thereby potentially aggravating the problem of stunted plant growth. However, methanotrophic activity in the root zone of LBC is not evenly distributed and varies with depth [10]. Therefore, root zone hypoxia caused by methanotrophic activity may also show uneven distribution. Although existing studies have discussed the importance of vegetation on LFG emission reductions, the interaction between plants and methanotrophs in LBCs is still poorly understood [10]. Therefore, the key objective of the current research is to fill these knowledge gaps and provide critical information on plant behavior in the presence of LFG, for professionals involved in the implementation of engineered biosystems, such as LBCs, for mitigating landfill CH_4_ emissions.

## 2. Materials and Methods

### 2.1. Study Location

The vegetated flow-through biofilter columns were set up outdoors at the Okotoks Eco-center research facility located in Okotoks, Alberta, Canada (50°42′58.78″ N and 113°57′0.94″ W). The facility is located in the prairie region of Canada, 1053 m above mean sea level and receives an annual average precipitation of 437 mm [24]. The climate is semi-arid and large temperature variations occur during summer and winter seasons, with mean minimum and maximum air temperatures ranging between −20.5 °C and 25.5 °C.

### 2.2. Flow-Through Vegetated Biofilter Columns

**Figure 1:**
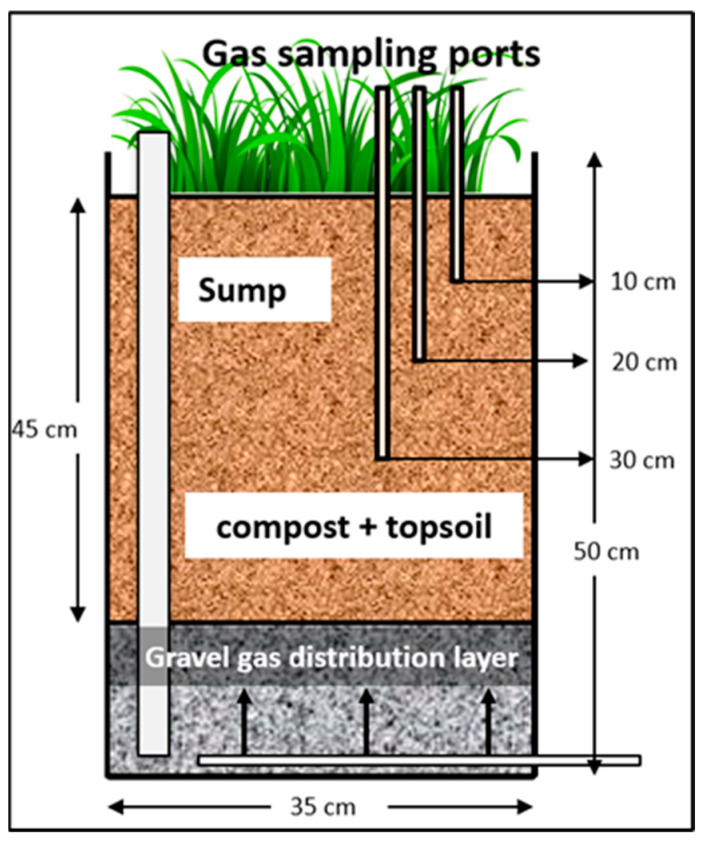
Schematic diagram of the constructed columns.

Eight high-density polyethylene (HDPE) flow-through biofilter columns of diameter 35 cm and height 50 cm were used for this study (see Figure 1). Each column was filled with a 5 cm layer of gravel at the bottom to ensure uniform gas distribution, followed by a 45 cm biofilter medium comprised of soil/compost mixture. To prevent potential clogging of the gravel layer, a non-woven geotextile layer was placed between the gravel and the biofilter medium. The biofilter medium was composed of 70% (*v/v*) topsoil sourced from a pile of topsoil (sandy lean clay) intended for use in a future landfill biocover at the Leduc regional landfill located in central Alberta, Canada, and 30% (*v/v*) residual compost obtained from the Edmonton waste management center (Edmonton, AB, Canada). The physical and chemical characteristics of the compost and soil are shown in Table 1. The thickness of the biofilter medium was chosen to be 45 cm based on previous research, which demonstrated that the majority of methane oxidation takes place within a narrow zone of approximately 20 cm, located about 10–15 cm below the top surface of the medium.

Three types of plants were chosen in this study: native grass (a blend of 25% Northern wheat grass—*Elymus lanceolatus*, 35% Awned wheat grass [*Elymus trachycaulaus*], 10% June grass [*Koeleria macrantha*], 25% Tufted hair grass [*Deschampsia caespitose*], and 5% Rough fescue [*Festuca hallii*]; Japanese millet (*Echinochloa esculenta*); and alfalfa (*Medicago sativa*). Each vegetated column was irrigated at a rate of 1 L/week after establishing the plants. The native grass blend is the most common type of vegetation used in LBCs; Japanese millet and alfalfa were selected in this study because they are cash crops and there is some interest among landfill operators to vegetate closed landfills with cash crops to ensure landfill space is used for beneficial purposes. Furthermore, with different root systems and potential for millet species plants to tolerate hypoxia because of the formation of aerenchyma, there is the possibility that each of the three selected species will produce different types of responses during exposure to CH_4_ gas.

### 2.3. Exposure of Vegetated Columns to Methane Gas

**Figure 2:**
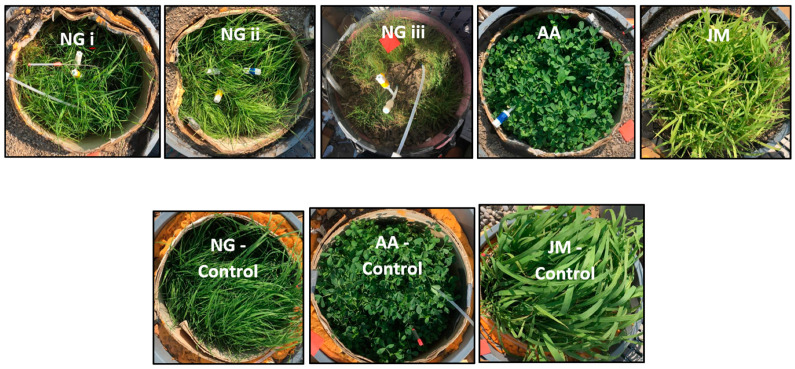
Experimental columns with different types of vegetative covers.

Five test columns and three control columns were used in this study. Three columns of native grass (triplicates), one column of Japanese millet, and one column of alfalfa were exposed to CH_4_ gas, while the remaining three columns were used as control columns with no gas exposure for each vegetation type, as shown in Figure 2. Details of all the eight columns are given in Table 2.

Over a 65-day period, natural gas containing 92% CH_4_ was introduced from the bottom of the gas-exposure columns at a pressure of 5 psi. Over this time period, the gas loading rates were gradually increased in six steps, ranging from 75.1 to 845.2 gCH_4_/m^2^/d. The loading rates were 75.1 gCH_4_/m^2^/d for the first 7 days, 173.1 gCH_4_/m^2^/d from day 7 to 27, 285.1 gCH_4_/m^2^/d from day 27 to 35, 437.9 gCH_4_/m^2^/d from day 35 to 41, 635.1 gCH_4_/m^2^/d from day 41 to 62, and 845.2 gCH_4_/m^2^/d from day 62 to 65. The ambient temperature varied from +9 °C to +26 °C during the experiment.

### 2.4. Determination of Plant Growth Characteristics

Plant growth characteristics, such as plant height and root length, were measured on day 35, day 50, and day 65 (at the end of the experiment). Plant height was measured from soil level to the terminal bud of a representative sample of plants and then averaged [25]. Similarly, root length was measured by collecting a representative sample of plants with minimum damage to the root systems and measuring the length from soil level to the tip of the root. At the conclusion of the column experiments on day 65, above-ground and below-ground dry biomass were measured by selecting sample plants from three areas (4 cm × 4 cm) of each column tested. To ensure the sample plants were representative of the population, the selection of these samples was performed in such a way as to include short, medium, and tall plants in each sample. The root systems were washed, cleaned, and separated from the rest of the plants. Both above-ground and below-ground plant tissue matter were separately dried in an oven at 60 °C for a minimum of 72 h and then weighed to calculate the plant above-ground and below-ground dry biomass density in g/cm^2^ [26].

The chlorophyll contents of the leaves of plants were determined on day 65 by treating 0.5 g of fresh leaf samples in an 80% acetone solution [27]. The optical densities of the total chlorophyll (α and β) content were determined at specific wavelengths of 645 and 663 nm using a UV-VIS spectrophotometer (Model: UV-2600, Shimadzu Corporation, Kyoto, Japan). The total chlorophyll content was calculated using the formula in [28],

(1) $$Chlorophyll\;a=\frac{12.3\;D_{663}-0.86\;D_{645}}{d\times 1000\times W}\times V$$

(2) $$Chlorophyll\;b=\frac{19.3\;D_{645}-3.6\;D_{663}}{d\times 1000\times W}\times V$$

(3) Total Chlorophyll content=Chlorophyll a+Chlorophyll b

where, V is the volume (mL) of acetone used for chlorophyll extraction, d is the light path length (cm), D663 is the absorbance at 663 nm, D645 is the absorbance at 645 nm, and W is the weight of the leaf used and the total chlorophyll content (a and b) in 1 g of sample leaf. To evaluate the significant differences in plant characteristics, such as height and root length at different growth stages between gas-exposed and control groups within each vegetation type, a two-way ANOVA was conducted. Additionally, *t*-tests were used to assess the significant differences between the gas-exposed and control groups in terms of vegetation characteristics, including above-ground and below-ground dry biomass, as well as chlorophyll content. The statistical analyses were performed using SigmaPlot 14.0 software from Systat Software Inc., and statistical significance was indicated where *p* < 0.05.

### 2.5. Determination of Gas Concentration Profiles in Columns

The gas concentration profiles of columns were measured on day 7, day 41, and day 65 by taking 5 mL samples from sampling ports located at 10, 20, and 30 cm depths using a luer-lock syringe with a two-way stainless-steel stop cock attached to a non-coring needle and then analyzing the samples for CH_4_, CO_2_, O_2_, and N_2_ using an SRI 8610C (SRI Instruments, Torrance, CA, USA) equipped with a thermal conductivity detector as described by La et al. [5].

### 2.6. Methane Oxidation Assessment

The surface CH_4_ emission fluxes of the test columns were measured using the static closed flux chamber method [29] to determine the rate and the efficiency of methane oxidation. The difference between the CH_4_ influx and the surface CH_4_ emission flux was used to calculate the rate of CH_4_ oxidation. The CH_4_ oxidation efficiency was calculated using the following equation as described by Powelson et al. [30],

(4) $$CH_{4}\;oxidation\;efficiency=\frac{\left(Flux_{loaded}-Flux_{emitted}\right)}{Flux_{loaded}}\left(100\right)$$

where, Flux_loaded_ and Flux_emitted_ are the CH_4_ inlet and surface emission fluxes in gCH_4_/m^2^/d, respectively.

After the completion of column experiments on day 65, undisturbed core soil samples were collected at depths between 15 to 25 cm from each column using a soil probe to perform batch oxidation kinetic experiments. Samples weighing 10–20 g were incubated in 250-mL airtight amber serum bottles with a headspace CH_4_ concentration of 10% [31]. The CH_4_ concentrations were then measured using gas chromatography at several time intervals until zero. The oxidation rates were then determined by plotting the change in headspace CH_4_ concentrations vs. time. The value of V_max_ [32] was obtained by using CH_4_ oxidation rates to produce substrate saturation curves as a function of initial headspace CH_4_ concentration. The data were then linearized with Eadie-Hofstee plots [31].

## 3. Results

In this section, first, the vegetation impacts during exposure of vegetated columns to CH_4_ gas are presented. Thereafter, the gas concentration profiles of the columns and CH_4_ oxidation results are presented in order to provide possible reasons for the vegetation impacts. The detailed discussion and interpretations of results, as well as the practical implications, are included in the next section.

### 3.1. Vegetation Growth Parameters with and without Gas Exposure

**Figure 3:**
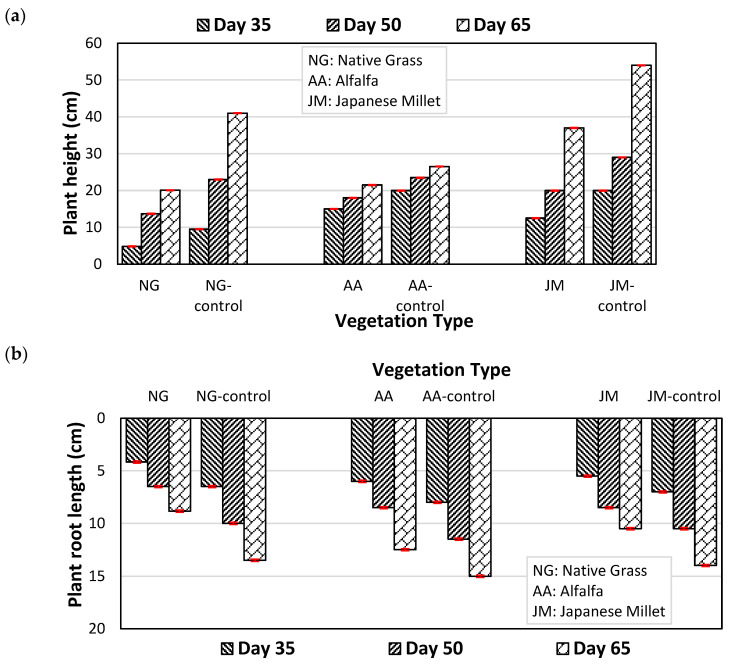

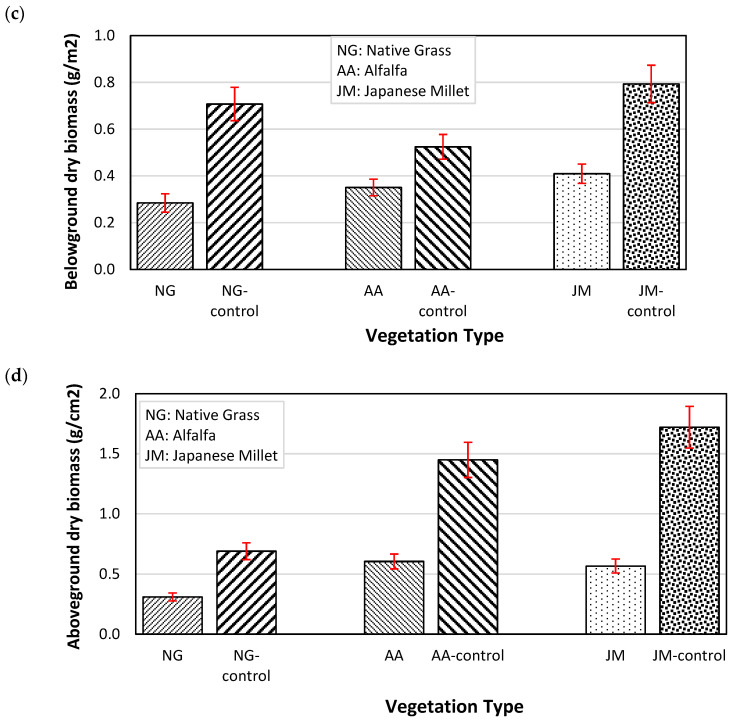
Comparison of vegetation characteristics under natural gas exposure and control (with no exposure); (**a**) Plant heights (**b**) Root lengths (**c**) Below-ground dry biomass densities (**d**) Above-ground dry biomass densities (NG: native grass, AA: alfalfa and JM: Japanese millet).

The growth parameters of the three plants; plant height, root length, and above-ground and below-ground plant dry biomass densities, with and without gas exposure, are shown in Figure 3a–d, respectively.

Figure 3a, representing the impact of CH_4_ gas exposure on plant height, shows that the highest plant height reductions were observed in native grass, with average reductions for the three replicates of 49.2%, 40.5%, and 61.0% in comparison with control columns, on day 35, day 50, and day 65, respectively. The lowest corresponding reductions of 25.0%, 23.4%, and 18.8% were observed for alfalfa, whereas the reductions for Japanese millet were 37.5%, 31.0%, and 31.5%. Similarly, as shown in Figure 3b, root length decreases of 35.9%, 35.0%, and 34.6% were observed for native grass after day 35, day 50, and day 65, respectively. Corresponding reductions were 25.0%, 26.1%, and 16.7% for alfalfa, and 21.4%, 19.0%, and 25.0% for Japanese millet. The results of the two-way ANOVA revealed statistically significant differences in plant height between the gas-exposed and control columns in both the native grass and alfalfa groups, with *p*-values of *p* = 0.049 for NG-gas vs. NG-control and *p* < 0.001 for AA-gas vs. AA-control. Similarly, the analyses showed statistically significant differences in root length between the gas-exposed and control columns in both vegetation types, with *p*-values of *p* < 0.001 for NG-gas vs. NG-control and *p* = 0.013 for AA-gas vs. AA-control.

As shown in Figure 3c,d, the below-ground dry biomass densities after 65 days of native grass, alfalfa, and Japanese millet were 0.28 (average of three replicates), 0.35, and 0.41 g/cm^2^, respectively, while the control groups had densities of 0.71, 0.52, and 0.79 g/cm^2^ for native grass, alfalfa, and Japanese millet, respectively. The above-ground dry biomass densities of gas-exposed native grass, alfalfa, and Japanese millet at the end of the experimental period (i.e., over the full 65 days) were 0.31 (average of three replicates), 0.60, and 0.57 g/cm^2^, respectively, while the dry biomass densities for the control groups were much higher, with values of 0.69, 1.45, and 1.72 g/cm^2^ for native grass, alfalfa, and Japanese millet, respectively. Consistent with previous findings, native grass exhibited the largest impact, while alfalfa had the least impact on both above- and below-ground dry biomass densities. The results of the student *T*-tests conducted on above-ground and below-ground dry biomass and chlorophyll content indicated no statistically significant differences between the growth of natural grass, alfalfa and Japanese millet in the gas-exposed and control columns.

**Figure 4:**
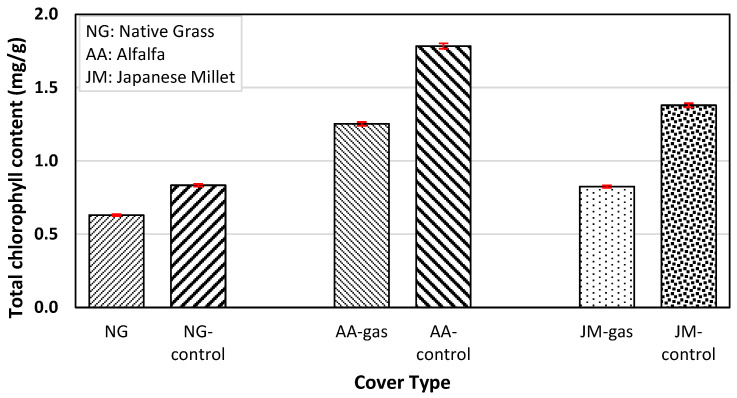
Chlorophyll content of different types of vegetation under two conditions: unexposed and exposed to natural gas (NG: native grass, AA: alfalfa and JM: Japanese millet).

The chlorophyll content of the three types of vegetation with gas exposure and control columns (i.e., with no gas exposure) is shown in Figure 4. The amount of total chlorophyll decreased in each type of vegetation exposed to CH_4_ gas when compared with the control columns, and native grass blend showed the least impact. Total chlorophyll content reductions of 24.4%, 29.8%, and 40.2% were observed for native grass (average of three replicates), alfalfa, and Japanese millet, respectively. Past research studies have shown that plants under stress develop chlorosis, or a yellowing of leaves, because of low O_2_ concentrations in the root zone [33,34]. Low O_2_ concentrations in the root zone inhibit chlorophyll biosynthesis, causing a decline in chlorophyll a, chlorophyll b, total chlorophyll, and carotenoid content [34]. This reduction in chlorophyll content under O_2_-deficit conditions can affect the rate of photosynthesis in plants, and therefore, plant growth as well [35].

### 3.2. Gas Concentration Profiles

**Figure 5:**
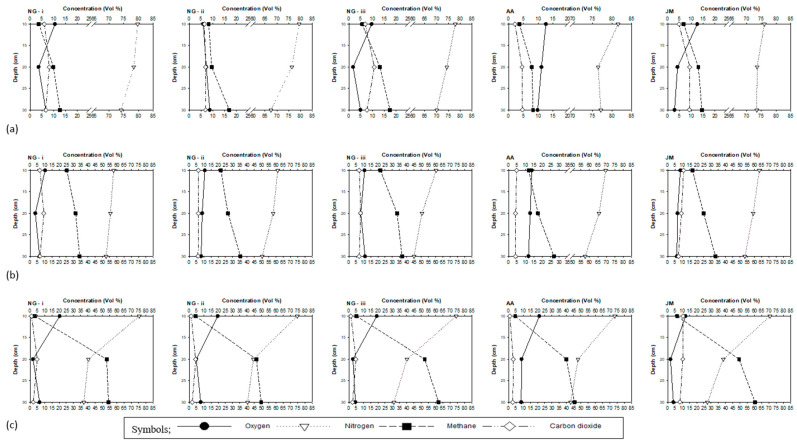
Depth profiles of the columns exposed to gas: NG-i, NG-ii, and NG-iii (native grass triplicates), AA—alfalfa, JM—Japanese millet, at different CH_4_ loading rates. (**a**) CH_4_ loading rate of 75.1 gCH_4_/m^2^/d on day 7, (**b**) CH_4_ loading rate of 437.9 gCH_4_/m^2^/d on day 41, (**c**) CH_4_ loading rate of 845.2 gCH_4_/m^2^/d on day 65.

The pore gas concentrations of N_2_, O_2_, CH_4_, and CO_2_ in the root zone at depths of 10, 20, and 30 cm from the top surface in each column are presented in Figure 5a–c, representing day 7, day 41, and day 65 at loading rates of 75.1 gCH_4_/m^2^/d, 437.9 gCH_4_/m^2^/d, and 845.2 gCH_4_/m^2^/d, respectively. These results indicate that the columns with native grass (NG) and Japanese millet (JM) contained less N_2_ and O_2_ than the column with alfalfa (AA). N_2_ and O_2_ concentrations along the depth are indicative of the level of soil aeration and air penetration [8], as well as the displacement of N_2_ and O_2_ by CH_4_ gas fed from the bottom of the columns. Furthermore, O_2_ consumption by methanotrophic bacteria will impact the O_2_ profiles. Whalen et al. pointed out that root morphology might play a major role in determining air penetration [36]. Root systems in alfalfa consist of deep taproots, while the root structures in native grass (and Japanese millet to a lesser extent) are shallow and fibrous. Such changes and root structure, as well as the differences in the rates of methane oxidation, may explain the distinct difference in gas profiles observed amongst the three vegetation species.

The highest concentrations of N_2_ among the gas-exposed columns were observed in column AA (or the column vegetated with alfalfa consisting of deep taproots). The values ranged from 81.5% to 77.2%, 69.6% to 57.5%, and 73.3% to 42.8% at loading rates of 75.1 gCH_4_/m^2^/d, 437.9 gCH_4_/m^2^/d, and 845.2 gCH_4_/m^2^/d, respectively, demonstrating that the displacement of N_2_ increases with increasing loading rates. Lower N_2_ concentrations were observed in JM and NG columns. The lowest N_2_ concentrations were observed in JM column at a loading rate of 845.2 gCH_4_/m^2^/d, with 70.6% at 10 cm, 38.4% at 20 cm, and 27.4% at 30 cm. The O_2_ concentrations in columns followed a similar trend in general, with AA column exhibiting O_2_ concentration values ranging from 12.6% to 9.8%, 13.7% to 11.8%, and 21.2% to 8.7% at loading rates of 75.1 gCH_4_/m^2^/d, 437.9 gCH_4_/m^2^/d, and 845.2 gCH_4_/m^2^/d, respectively. Similar to N_2_ concentrations, the lowest O_2_ concentrations were observed in JM column at a loading rate of 845.2 gCH_4_/m^2^/d, with 12.3%, 1.9%, and 3.9% at depths 10, 20, and 30 cm, respectively. The results from this study are similar to those reported by Whalen et al. [36]. They reported that lysimeters with grass had lower aeration, indicated by low O_2_, than a lysimeter with alfalfa and grass, indicated by high O_2_ levels.

The CO_2_ profiles showed a different trend simply because CO_2_ concentrations are not directly impacted by root morphology and air penetration, but by the level of root respiration (if any) and methane oxidation occurring in the root zone. At the loading rate of 75.1 gCH_4_/m^2^/d, the highest concentrations of CO_2_ were observed in column NG-iii with concentrations of 6.9%, 10.8%, and 7.7%, at depths 10, 20, and 30 cm, respectively. These concentration results are indicative of the occurrence of methane oxidation in the 20 cm depth range. Typically, the majority of methane oxidation in columns occurs at depths between 10 to 30 cm below the column surface. The CO_2_ concentrations in column AA were low, with concentrations ranging from 2.1% to 4.7%, 3.9% to 4.8%, and 0.9% to 3.1% at loading rates of 75.1 gCH_4_/m^2^/d, 437.9 gCH_4_/m^2^/d, and 845.2 gCH_4_/m^2^/d, respectively. In column JM, at a loading rate of 845.2 gCH_4_/m^2^/d, the CO_2_ concentrations were 10.5%, 10.4%, and 8.4%, each at depths of 10, 20, and 30 cm, respectively.

The CH_4_ concentrations in vegetated columns are determined by the loading rates and methane oxidation, with high CH_4_ concentrations typically observed at a depth of 30 cm. At the lowest CH_4_ loading rate of 75.1 gCH_4_/m^2^/d, the CH_4_ concentrations ranged from 3.7% to 12.6% in NG-i, 8.2% to 16.9% in NG-ii, 5.6% to 17.4% in NG-iii, 3.7% to 8.3% in AA, and 6.7% to 14.4% in JM at depths between 10 and 30 cm. During the experiment, the highest CH_4_ concentration of 61.9% was observed in the natural gas column NG-iii exhibited at 30 cm depth and at the highest loading rate of 845.2 gCH_4_/m^2^/d. Although this column showed a relatively high oxidation rate, most oxidation seemed to have taken place between the depths of 10 to 20 cm. In general, the columns vegetated with native grass showed the highest CH_4_ concentrations throughout the experiment, and the lowest CH_4_ concentrations were exhibited by column AA. For example, at the loading rate of 437.9 gCH_4_/m^2^/d, the CH_4_ concentration values ranged from 25.3% to 34.2% in NG-i, 21.8% to 35.3% in NG-ii, and 21.8% to 36.7% in NG-iii at depths of 10 to 30 cm, whereas, the values for column AA ranged from 11.9% to 26.8% at depths of 10 to 30 cm. This observation is attributed to alfalfa plants having deep taproots, which allows an escape of CH_4_ gas from deeper parts of the columns via the macropores created by the deep roots.

Both CH_4_ and CO_2_ may exhibit negative impacts on the growth of a plant; CH_4_, although not a phytotoxin, causes displacement of O_2_ when present in high concentrations [20]. Previous studies have shown that high CO_2_ concentrations in the root zone of a plant can considerably hinder its growth [37]. Roots under hypoxic stress may transmit signals to the leaves, limiting leaf growth and substrate transportation to the root system, and promoting the accumulation of certain adverse compounds in the leaves [38,39].

Root morphology might play a major role in the performance of the system: roots take up water, increasing permeability (i.e., increasing penetration of N_2_ and O_2_). Root systems in alfalfa consist of deep taproots that can extend to 35 cm, while the root systems in grass are shallow (root mat) and only extend to about 20 cm [24]. Alfalfa plants used in our study had taproots, while the native grass and Japanese millet had fibrous root structures, explaining the distinct difference in gas profiles amongst the different vegetation species.

### 3.3. Methane Oxidation in Vegetated Columns

**Figure 6:**
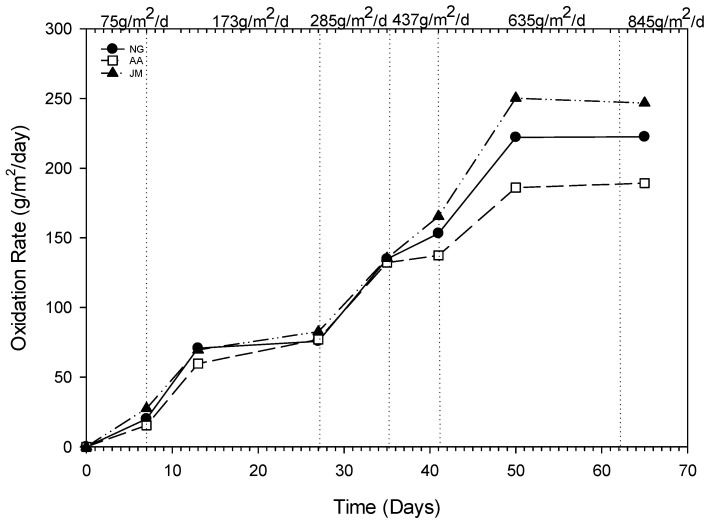
Time-dependent methane oxidation rates (NG: native grass (triplicates indicated as i, ii and iii), AA: alfalfa and JM: Japanese millet).

The average CH_4_ oxidation rates and oxidation efficiencies at each flux rate are shown in Table 3. The time and flux dependent oxidation rates for columns with different types of vegetation (NG—average from native grass triplicates of NG-i, NG-ii and NG-iii), AA (alfalfa), and JM (Japanese millet) are presented in Figure 6.

Although the CH_4_ oxidation efficiency fluctuated, the CH_4_ oxidation rate increased with an increase in CH_4_ influx in all columns. The column JM, with Japanese millet, exhibited the highest methane oxidation efficiencies and oxidation rates throughout the experiment. The highest average methane oxidation efficiency was observed in column JM with a value of 47.5% at the loading rate of 285.1 gCH_4_/m^2^/d. In column JM, CH_4_ oxidation rate increased from 27.5 to 246.8 gCH_4_/m^2^/d when the CH_4_ loading rate increased from 75.1 to 845.2 gCH_4_/m^2^/d. The corresponding methane oxidation rate values for columns AA and NG (average of triplicates) were 15.4 to 189.3 gCH_4_/m^2^/d and 20 to 222.6 gCH_4_/m^2^/d, respectively. The oxidation rates observed in this study were much higher than the rates observed by other researchers with vegetated columns. In a study by Bohn et al., where the overall maximum loading rate was 89.6 gCH_4_/m^2^/d, the three vegetated columns showed maximum oxidation rates of 89.6 gCH_4_/m^2^/d, 62.4 gCH_4_/m^2^/d, and 36.8 gCH_4_/m^2^/d for the columns with grass mixture, Canadian goldenrod, and leguminous mixture, respectively [23]. They observed an increase in O_2_ diffusion into the granular medium that potentially increased methane oxidation. Similar to our study, the extended plant roots, especially JM columns vegetated with Japanese millet, may have increased the air-filled capacity and O_2_ diffusion into the root zone.

The calculated V_max_ values related to methane oxidation in the columns are presented in Table 4. The V_max_ values represent the viable methanotrophic bacterial populations present in the soil core samples obtained from each column on day 65. The methanotrophic activity V_max_ values of the gas-exposed columns, from highest (6.64 µmol/g dw/h) to lowest (2.92 µmol/g dw/h), were as follows: JM > NG > AA. This data corresponds well with the calculated CH_4_ oxidation rates presented in Table 3, with the highest oxidation rates being observed in JM, while the lowest were observed in AA. The V_max_ values obtained in this study are comparable to values of 2.8 µmol/g dw/h [40] and 7.7 µmol/g dw/h [31] obtained in other studies conducted on the methanotrophic activities of cover soil material in the presence of CH_4_ [31].

## 4. Discussion and Interpretation of Results

### 4.1. Oxygen Gas Concentrations and Vegetation Impacts

The gas concentration profiles of columns presented in Figure 5a–c, representing exposure to the flux rates of 75.1 to 845 gCH_4_/m^2^/d, show that O_2_ concentrations in almost all vegetated columns are below 10%. Low levels of O_2_ affect the oxygen-dependent reactions within plants [41]. An O_2_ level above 10% in the soil-gas phase of the root zone is needed for the growth of most plants, and an O_2_ level below 10% causes hypoxia in plants [19,22]. It is known that root zone hypoxia stress in plants triggers the formation of reactive oxygen species, such as superoxide radicals, hydroxyl radicals, and hydrogen peroxide, which causes damage to DNA, membranes, and proteins [42], resulting in chlorosis, necrosis, defoliation, stunted growth, and root damage [41,43]. Plant cells under hypoxic conditions synthesize higher concentrations of ethylene, a plant hormone, which then induces the formation of lysingenous aerenchyma—gas filled spaces resulting from lysis and the death of cells in the root cortex [44,45].

**Figure 7:**
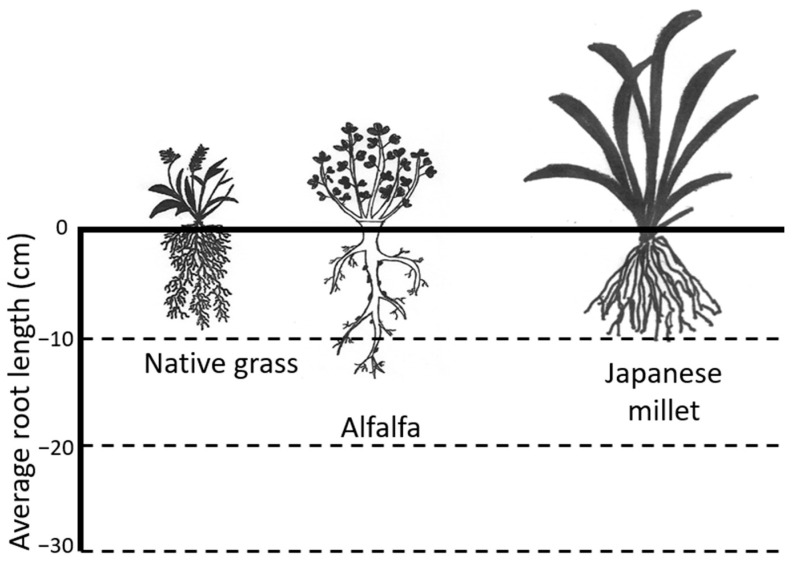
Schematic diagram of plants used in the study.

In the current study, the lowest O_2_ concentrations were observed with native grass columns (average of NG-i, NG-ii and NG-iii), and consequently, these columns exhibited the highest impact on vegetation. For example, at the relatively high flux rate of 845 gCH_4_/m^2^/d, the O_2_ concentrations in the 20 cm region were well below 5%, and there was a large reduction of 51% in plant height and 35% in root length in the native grass blend in comparison with the plants in the control column. The corresponding values for Japanese millet were a 31.5% reduction in plant height and 25% in root length, and in alfalfa were a 19% reduction in plant height and 17% in root length. Whalen et al. [36] pointed out that root morphology might play a major role in the plant behavior: roots take up water, increasing permeability, thereby increasing the penetration of N_2_ and O_2_. Alfalfa root systems are characterized by deep taproots that can extend up to 50–60 cm, creating preferential pathways for gas, water, and nutrients. In contrast, grass root systems form a shallow root mat that only extends 20–30 cm below the surface [24]. The alfalfa plants used in our study also had taproots, whereas the native grass and Japanese millet had fibrous root structures, as depicted in Figure 7. This difference in root structures may explain the distinct differences in gas profiles observed among the different vegetation species. Some plants may undergo a physical and morphological adaptation known as “Aerenchyma formation” to overcome hypoxic conditions in soil [46,47]. Aerenchyma are a form of cells that ensure the survival of certain plants that are under extremely O_2_ deficient conditions and act as conduits that supply O_2_ to the roots [48,49]. It appears that Japanese millet plants have the capability to tolerate hypoxia to some extent because of the supply of O_2_ to the roots via the lysigenous aerenchyma along the adventitious root [50].

### 4.2. Methane Oxidation and Vegetation Impacts

Methane oxidation by methanotrophs could impact plant growth in two ways; by consuming O_2_ in competition with plants and by producing CO_2_ that could impact plant growth. In experimental soil columns, most methanotrophic activity occurs in a narrow range about 15–40 cm below surface, known as the zone of methane oxidation [51]. However, in vegetated columns, this zone could extend beyond this range because of the changes in porosity in the presence of plant roots [10]. As a result of the presence of roots, the zone of methane oxidation is not highly pronounced in our vegetated columns. Nevertheless, the concentration profiles of columns presented in Figure 5a–c show high levels of CO_2_ in the 5 to 10% range, in almost all vegetated columns. The presence of CO_2_ is the result of methane oxidation by methanotrophs that converts CH_4_ to CO_2_ and water.

Past research studies have shown that when CO_2_ exceeds 20% volume in the soil pore space, vegetation could be negatively impacted [18,52]. Even lower CO_2_ levels of 2% (by volume) in the root zone of pea plants (*Pisum sativum* L.) have been shown to reduce the growth of the roots by 80% [37]. Considering almost all of our columns exhibited greater than 2% CO_2_ (by volume), it is not surprising to observe impacts on root growth of all plants tested.

Another consequence of methane oxidation in columns is the consumption of O_2_ in the root zone by methanotrophs decreasing the pore O_2_ available for plants. As expected, CH_4_ oxidation rates increased with an increase in CH_4_ influx, and therefore, the highest oxidation rates were observed at the highest methane flux rate of 845 gCH_4_/m^2^/d. The columns with Japanese millet exhibited the highest methane oxidation rate of 250.2 gCH_4_/m^2^/d. The corresponding methane oxidation rates were 222.5 gCH_4_/m^2^/d for column NG, and 189.3 gCH_4_/m^2^/d for alfalfa. Since these values were within the experimental error for the column experiments, definitive conclusions cannot be made as to the presence of a direct correlation between methane oxidation rate and the occurrence of hypoxia in columns, which is the primary cause of vegetation impacts. Furthermore, we observed cumulative negative impacts on vegetation from the reduction of O_2_ in the root zone, as well as the increase in CO_2_ levels because of methane oxidation. One other confounding factor is the increased diffusion into the root zone of O_2_ from the atmosphere because of the macropores created by the growth of root systems over time [10]. This phenomenon of increased O_2_ diffusion into the root zone tends to counterbalance the decrease in O_2_ because of methane oxidation and high loading rates of gas.

## 5. Conclusions

Considering past research that has shown vegetation on landfills could be impacted because of potential hypoxia in instances where (1) generated landfill gas displaces oxygen in the root zone of the plants, and (2) plants compete for oxygen with methanotrophic bacteria present in the root zone, this study was conducted to investigate the effects of methane exposure on three types of potential landfill vegetation, native grass, alfalfa, and Japanese millet. The study included conducting experiments outdoors to determine time-dependent changes in gas concentration profiles, methane oxidation rates, and efficiencies and kinetics, as well the determination of changes to plant height, root length, dry mass density, and chlorophyll content during exposure to methane flux rates ranging from 75 gCH_4_/m^2^/d to 845 gCH_4_/m^2^/d. Although definitive results could not be obtained due to constraints associated with conducting outdoor experiments under semi-controlled conditions, practical implications of the experimental results were clear. Plant growth in landfill biocover systems will be somewhat impacted even at 75 gCH_4_/m^2^/d of methane fluxes, representative of typical average flux rates expected at waste cells accepting biodegradable organic waste. At higher flux rates representative of hotspots on a landfill surface, the impacts will be greater, but extremely high flux rates, greater than 845 gCH_4_/m^2^/d, are required to completely kill off vegetation.

## Figures and Tables

**Table 1:** Characteristics of column construction materials.

Parameters	Units	Topsoil	Residual Compost
Moisture content	%	22.74	28.77
Organic matter	%	5.89	49.53
Dry bulk density	(g/cm^3^)	1.21	0.95
Field Capacity	%	45.06	164.94
C/N	ratio	15.30	10.57

**Table 2:** Vegetation type and gas exposure details of columns.

Column ID	Type of Vegetation	Natural Gas Exposure
NG-i	Native grass	+
NG-ii	Native grass	+
NG-iii	Native grass	+
AA	Alfalfa	+
JM	Japanese Millet	+
NG-control	Native grass	−
AA-control	Alfalfa	−
JM-control	Japanese Millet	−

(+) sign indicates exposure to methane gas and (−) indicates control groups.

**Table 3:** Average oxidation rate and oxidation efficiencies of columns.

Loading Rate (g/m^2^/d)	Oxidation Rate (gCH_4_/m^2^/d)			Oxidation Efficiency (%)		
	NG (Average of i, ii and iii)	AA	JM	NG	AA	JM
75.1	20.0	15.4	27.5	26.6	20.5	36.6
173.1	73.2	68.4	76.1	42.3	39.5	43.9
285.1	134.8	132.3	135.4	47.3	46.4	47.5
437.9	153.2	137.5	165.5	35	31.4	37.8
635.1	222.1	186.1	250.2	34.9	29.3	39.4
845.2	222.5	189.3	246.8	26.3	22.4	29.2

**Table 4:** Vmax values for columns at depths of 15–25 cm.

Kinetic Parameter	NG	AA	JM	NG-con	AA-con	JM-con
Vmax (µmol/g dw/h)	3.97	2.92	6.64	0.22	0.24	0.51

## Data Availability

All data, models, or code that support the findings of this study are available from the corresponding author upon reasonable request.

## References

[1] Hunte C., Hettiaratchi J.P.A., Hettiarachchi H., Meegoda J.N. (2011). Determination of Waste Properties from Settlement Behaviour of a Full Scale Waste Cell Operated as a Landfill Bioreactor.

[2] Xiaoli C., Xin Z., Ziyang L., Shimaoka T., Nakayama H., Xianyan C., Youcai Z. (2011). Characteristics of vegetation and its relationship with landfill gas in closed landfill. Biomass Bioenergy.

[3] Bogner J.E., Spokas K.A., Burton E.A. (1999). Temporal variations in greenhouse gas emissions at a midlatitude landfill. J. Environ. Qual..

[4] Kightley D., Nedwell D.B., Cooper M. (1995). Capacity for methane oxidation in landfill cover soils measured in laboratory-scale soil microcosms. Appl. Environ. Microbiol..

[5] La H., Hettiaratchi J.P.A., Achari G., Verbeke T.J., Dunfield P.F. (2018). Biofiltration of methane using hybrid mixtures of biochar, lava rock and compost. Environ. Pollut..

[6] Tao Z., Shi W., Liu Y., Chai X. (2020). Temporal variation of vegetation at two operating landfills and its implications for landfill phytoremediation. Environ. Technol..

[7] Khapre A., Kumar S., Rajasekaran C. (2019). Phytocapping: An alternate cover option for municipal solid waste landfills. Environ. Technol..

[8] Reichenauer T.G., Watzinger A., Riesing J., Gerzabek M.H. (2011). Impact of different plants on the gas profile of a landfill cover. Waste Manag..

[9] Stralis-Pavese N., Sessitsch A., Weilharter A., Reichenauer T., Riesing J., Csontos J., Murrell J.C., Bodrossy L. (2004). Optimization of diagnostic microarray for application in analysing landfill methanotroph communities under different plant covers. Environ. Microbiol..

[10] Attalage D.S., Hettiaratchi P.A., Jayasinghe P., Dunfield P.F., Smirnova A.V., Rathnavibushana U.K., Erkmen M., Kumar S. (2022). Field study on the effect of vegetation on the performance of soil methanotrophy-based engineered systems—Column experiments. Soil Biol. Biochem..

[11] Reay D.S., Nedwell D.B., McNamara N., Ineson P. (2005). Effect of tree species on methane and ammonium oxidation capacity in forest soils. Soil Biol. Biochem..

[12] Chan Y.S.G., Chu L.M., Wong M.H. (1997). Influence of landfill factors on plants and soil fauna—An ecological perspective. Environ. Pollut..

[13] Wong M.H., Yu C.T. (1989). Monitoring of gin drinkers’ bay landfill, Hong Kong: II. Gas contents, soil properties, and vegetation performance on the side slope. Environ. Manag..

[14] Wong M.H., Yu C.T. (1989). Monitoring of gin drinkers’ bay landfill, Hong Kong: I. landfill gas on top of the landfill. Environ. Manag..

[15] Leone I.A., Flower F.B., Arthur J.J., Gilman E.F. (1977). Damage to New Jersey crops by landfill gases. Plant Dis. Rep..

[16] Gilman E.F., Leone I.A., Flower F.B. (1981). The adaptability of 19 woody species in vegetating a former sanitary landfill. For. Sci..

[17] Wong M.H., Cheung Y.H., Cheung C.L. (1983). The effects of ammonia and ethylene oxide in animal manure and sewage sludge on the seed germination and root elongation of brassica parachinensis. Environ. Pollution. Ser. A Ecol. Biol..

[18] Gendebien A., Pauwels M., Ledrut-Damanet M.J., Nyns E.J., Willumsen H.C., Butson J., Fabry R., Ferrero G.L. (1992). Potential Landfill Gas Damages to Vegetation. Landfill Gas from Environment to Energy.

[19] Kozlowski T.T. (1985). Soil aeration, flooding, and tree growth. J. Arboric..

[20] Nagendran R., Selvam A., Joseph K., Chiemchaisri C. (2006). Phytoremediation and rehabilitation of municipal solid waste landfills and dumpsites: A brief review. Waste Manag..

[21] Gilman E.F., Leone I.A., Flower F.B. (1982). Influence of soil gas contamination on tree root growth. Plant Soil.

[22] Danielson R.E. (1974). Physical edaphology—The physics of irrigated and nonirrigated soils. Soil Sci. Soc. Am. J..

[23] Bohn S., Brunke P., Gebert J., Jager J. (2011). Improving the aeration of critical fine-grained landfill top cover material by vegetation to increase the microbial methane oxidation efficiency. Waste Manag..

[24] Jalilzadeh H. (2019). Field Performance and Water Balance Predictions of Evapotranspirative Landfill Biocovers. Master’s Thesis.

[25] Adenipekun C.O., Oyetunji O.J., Kassim L.S. (2008). Effect of spent engine oil on the growth parameters and chlorophyll content of corchorus olitorius linn. Environmentalist.

[26] Cornelissen J.H.C., Lavorel S., Garnier E., Díaz S., Buchmann N., Gurvich D.E., Reich P.B., ter Steege H., Morgan H.D., van der Heijden M.G.A. (2003). A handbook of protocols for standardised and easy measurement of plant functional traits worldwide. Aust. J. Bot..

[27] Singh S.N., Rao D.N. (1981). Certain responses of wheat plants to cement dust pollution. Environ. Pollut. Ser. A Ecol. Biol..

[28] Maclachlan S., Zalik S. (1963). Plastid structure, chlorophyll concentration, and free amino acid composition of a chlorophyll mutant of barley. Can. J. Bot..

[29] Pihlatie M.K., Christiansen J.R., Aaltonen H., Korhonen J.F.J., Nordbo A., Rasilo T., Benanti G., Giebels M., Helmy M., Sheehy J. (2013). Comparison of static chambers to measure CH4 emissions from soils. Agric. Meteorol..

[30] Powelson D.K., Chanton J., Abichou T., Morales J. (2006). Methane oxidation in water-spreading and compost biofilters. Waste Manag. Res. J. A Sustain. Circ. Econ..

[31] Pokhrel D., Hettiaratchi J., Steele M. (2016). Methane oxidation prediction curves of soil at different organic contents. Curr. Environ. Manag..

[32] Pokhrel D. (2006). Compost Based Biocap Performance. Ph.D, Thesis.

[33] Flower F.B., Gilman E.F., Leone I.A. (1981). Landfill gas, what it does to trees and how its injurious effects may be prevented. J. Arboric..

[34] Mi Y., Ma X., Chen S. (2013). Resistant evaluation of kiwifruit rootstocks to root zone hypoxia stress. Am. J. Plant Sci..

[35] Cao F.L., Conner W.H. (1999). Selection of flood-tolerant populus deltoides clones for reforestation projects in China. For. Ecol. Manag..

[36] Whalen S.C., Reeburgh W.S., Sandbeck K.A. (1990). Rapid methane oxidation in a landfill cover soil. Appl. Environ. Microbiol..

[37] Stolwijk J.A.J., Thimann K.V. (1957). On the uptake of carbon dioxide and bicarbonate by roots, and its influence on growth. Plant Physiol..

[38] Jackson M.B., Hall K.C. (1987). Early stomatal closure in waterlogged pea plants is mediated by abscisic acid in the absence of foliar water deficits. Plant Cell Environ..

[39] Milligan S.P., Dale J.E. (1988). The effects of root treatments on growth of the primary leaves of phaseolus Vulgaris L.: General features. N. Phytol..

[40] Chiemchaisri W., Visvanathan C., Wu J.S. (2001). Biological activities of methane oxidation in tropical landfill cover soils. J. Solid Waste Technol. Manag..

[41] Hanslin H.M., Sæbø A., Bergersen O. (2005). Estimation of oxygen concentration in the soil gas phase beneath compost mulch by means of a simple method. Urban For. Urban Green.

[42] Geigenberger P. (2003). Response of plant metabolism to too little oxygen. Curr. Opin. Plant Biol..

[43] Boru G., Vantoai T., Alves J., Hua D., Knee M. (2003). Responses of soybean to oxygen deficiency and elevated root-zone carbon dioxide concentration. Ann. Bot..

[44] Drew M.C. (1997). Oxygen deficiency and root metabolism: Injury and acclimation under hypoxia and anoxia. Annu. Rev. Plant Biol..

[45] Evans D.E. (2004). Aerenchyma Formation. N. Phytol..

[46] Joshi R., Kumar P. (2012). Lysigenous aerenchyma formation involves non-apoptotic programmed cell death in rice (Oryza Sativa L.) roots. Physiol. Mol. Biol. Plants.

[47] Jiang Z., Song X.F., Zhou Z.Q., Wang L.K., Li J.W., Deng X.Y., Fan H.Y. (2010). Aerenchyma formation: Programmed cell death in adventitious roots of winter wheat (Triticum aestivum) under waterlogging. Funct. Plant Biol..

[48] Colmer T.D. (2003). Long-distance transport of gases in plants: A perspective on internal aeration and radial oxygen loss from roots. Plant Cell Environ..

[49] Yamauchi T., Tanaka A., Mori H., Takamure I., Kato K., Nakazono M. (2016). Ethylene-dependent aerenchyma formation in adventitious roots is regulated differently in rice and maize. Plant Cell Environ..

[50] Matsuura A., Kato Y., Suzuki T., Murata K., An P. (2022). Hypoxia tolerance of four millet species is attributable to constitutive aerenchyma formation and root hair development of adventitious roots. Plant Prod. Sci..

[51] Visvanathan C., Pokhrel D., Cheimchaisri W., Hettiaratchi J.P.A., Wu J.S. (1999). Methanotrophic activities in tropical landfill cover soils: Effects of temperature, moisture content and methane concentration. Waste Manag. Res..

[52] Ponnamperuma F.N. (1972). The chemistry of submerged soils. Adv. Agron..

